# Considerations for the design of nutrition-sensitive production programmes in rural South Africa

**DOI:** 10.1186/s12889-020-09445-3

**Published:** 2020-09-10

**Authors:** S. L. Hendriks, A. Viljoen, D. Marais, F. A. M. Wenhold, A. M. McIntyre, M. S. Ngidi, J. G. Annandale, M. Kalaba, D. Stewart

**Affiliations:** 1 Department of Agricultural Economics, Extension and Rural Development, University of Pretoria, PBag X01, Hatfield, Pretoria, 0028 South Africa; 2 Department of Consumer Science, University of Pretoria, PBag X01, Hatfield, Pretoria, 0028 South Africa; 3 Department of Plant and Soil Sciences, University of Pretoria, PBag X01, Hatfield, Pretoria, 0028 South Africa; 4 Department of Human Nutrition, University of Pretoria, X323, Arcadia, Pretoria, 0007 South Africa; 5 Department of Agricultural Economics, Extension and Rural Development, University of Pretoria, PBag X01, Hatfield, Pretoria, 0028 South Africa; 6 Department of Agricultural Extension and Rural Resource Management, School of Agricultural, Earth and Environmental Sciences, University of KwaZulu-Natal, Pietermaritzburg, PBag01, Scottsville, 3209 South Africa; 7 Department of Plant and Soil Sciences, University of Pretoria, PBag X01, Hatfield, Pretoria, 0028 South Africa; 8 Department of Agricultural Economics, Extension and Rural Development, University of Pretoria, PBag X01, Hatfield, Pretoria, 0028 South Africa; 9 Lima Rural Development Foundation, 2 Forrester’s Lane, Pietermaritzburg, 3201 South Africa

**Keywords:** Nutrition-sensitive agriculture, Agricultural production, Dietary diversity, Irrigation, Food security

## Abstract

**Background:**

Very little has been researched about the efficacy, effectiveness, feasibility, sustainability and impact of food-based approaches on the diets and nutritional status of populations at risk of hunger and food insecurity. This study contributes knowledge about the impact of food-based approaches on the diets of populations at risk of hunger and food insecurity in four of the poorest rural communities in South Africa. The study investigated the consumption and production patterns of rural households (278 in summer and 280 in winter) in four sites in the poorest municipalities in South Africa.

**Methods:**

A multistage stratified random sampling technique was applied to identify the communities and sample households for the quantitative survey and qualitative assessments. Qualitative and quantitative data were collected between 2013 and 2015 through focus group discussions (FGDs), key informant interviews and the two-round panel survey to cover both the summer and winter seasons at each site.

**Results:**

Home gardening led to a significant positive increase in the consumption of white roots and tubers, dark green leafy vegetables, orange-coloured fruit and other fruit in the 24 h prior to the survey. Participation in a community garden led to significant increases in the consumption of dark green leafy vegetables and other vegetables. School gardening did not demonstrate any statistical relationships with the consumption of foods from the crop-related food groups. Crop production improved dietary diversity. Selling produce and irrigation showed a stronger improvement in dietary diversity. Seasonality affected the availability of fresh fruit and vegetables for home consumption in winter.

**Conclusions:**

Producing beyond that solely for home consumption has greater benefits for dietary diversity and a consumption-smoothing effect during the post-harvest period. Politicians and the scientific community should recognise the role that household and small-scale crop production plays in supporting household consumption and the provision of essential micronutrients despite constraints and disincentives. Production and education programmes should focus on strengthening existing good consumption patterns and promoting the consumption of foods that can improve dietary diversity.

## Background

Nutrition-sensitive agriculture or the production of foods with high nutrient densities (such as dairy, fish, fruit, meat and vegetables), is recognised as a pathway to improved nutrition, increasing the availability and access to nutritious foods and creating opportunities for generating income from the sale of produce [1]. Recent attention to nutrition-sensitive agriculture and food systems calls for changes in producer support programmes, the research and development system and extension practice to broaden their focus beyond the production of staple crops to the promotion and support of agricultural systems that increase the supply of nutrient-dense foods at household level and in the food system in general.

Despite significant policy attention to subsistence and smallholder production in South Africa, there is a pervasive opinion among South African policy makers and researchers alike that households in the country’s rural areas are not actively engaged in agricultural production. Many claim that we are in a period of deagrarianisation (reorientation of livelihoods away from agrarian patterns), claiming that poor households can more easily access cheap food (predominantly staples) from the expanding supermarket network [2–4]. Others claim that the reduction in government support to former the homelands has been a disincentive to production and perpetuates neglect [4]. It is also reported that the availability of staple foods in supermarkets, climate variability, erratic rainfall patterns, an aging rural population, disinterest in agriculture by the younger generation and the widespread access to social grants all act as disincentives to production in these areas [5].

Yet, a systematic review of 169 subnational food insecurity studies conducted in the post-apartheid period in South Africa between 1994 and 2014 by Misselhorn and Hendriks [5], found that food gardens generally play an important part in improving diet quality through the inclusion of fresh fruit and vegetables–even if only seasonally. They can also contribute to building knowledge about healthy dietary choices, build social capital and contribute to community development through enhanced networks and cooperation. However, the role of agricultural production in food security in South Africa’s poorest communities has not been rigorously investigated [5]. While there is not much evidence of widespread starvation and acute under-nutrition in the country, there is clear evidence of multiple forms of deprivation [6]. Devereux and Waidler [7] claim that food security has certainly improved for most South Africans post-1994, but the nutrition status of children has stagnated or only improved marginally. National surveys have found significant levels of self-reported hunger, a widespread manifestation of ‘hidden hunger’ or micronutrient deficiencies and the co-existence of overweight and obesity alongside hidden hunger and child undernourishment [8–10]. Despite a multitude of state, private sector and NGO-funded food security programmes, stunting levels (an indication of chronic food insecurity) in South Africa increased over the Millennium Development Goal (MDG) period [11, 12]. The increasing incidence of overweight among women and children raises alarm. Both the incidence of underweight and overweight indicate severe inadequacies related to the diets of South Africans.

Despite wide recognition that producing one’s own food increases access to nutritious foods and can provide income to purchase other foods and non-food essentials [1], very little has been researched or is known internationally about the efficacy, effectiveness, feasibility, sustainability and impact of food-based approaches on the diets and nutritional status of populations at risk of hunger and food insecurity [13]. Ruel et al. [1] report that despite growing commitment from governments, donor agencies and development organisations to supporting nutrition-sensitive agriculture to achieve their development goals, empirical evidence on agriculture’s contribution to nutrition and how it can be enhanced is still weak. Nutrition-sensitive interventions or programs are those that address the underlying determinants of nutrition and development and incorporate specific nutrition goals and actions [14].

A very recent review of research findings published over the past two decades by Ruel et al. [1] has reported that evidence on what and how agriculture can contribute to nutrition is extremely scant. Ruel et al. [1], report that the available studies found that agricultural development programs that promote production diversity, micronutrient-rich crops (including biofortified crops), dairy or small animal rearing can improve the production and consumption of targeted commodities. Some evidence existed that these improvements lead to increases in dietary diversity at the household. Similarly, a 2012 review of current and planned research on agriculture for improved nutrition projects funded by the United Kingdom’s Department for International Development (DFID) around the world found that only 43 of a 100 fully-mapped projects – under half – measured or even considered nutritional status [15]. A 2011 international review of 30 years of agricultural projects under the United States Agency for International Development’s Infant and Young Child Nutrition Programme [16] showed that agricultural projects significantly improve household incomes and access to food, but the short-term impact of these increases does not have the same level of significance in terms of improving the nutritional status of young children in poor communities. Where improvements are seen, these are usually improvements in dietary intakes (especially where vitamin A-rich foods are produced), rather than improvements in anthropometry [16]. This is most likely due to the fact that food-based and agricultural strategies targeted at food insecure populations do not significantly improve the macronutrient (energy, carbohydrate, protein and fat) intakes of nutritionally at-risk individuals - an essential element to overcoming chronic food insecurity.

Sibhatu and Qaim [17] report little is known about how much subsistence agriculture actually contributes to household diets and how this contribution changes seasonally. These authors point out that most studies of the pathways for improving food security through agricultural production are based on data from cross-sectional surveys that are carried out once-off at one particular time of the year. Yet, the diets of poor rural households vary seasonally. While studies of dietary adequacy and nutrition outcomes have examined the s\effects of seasonal outcomes of agricultural projects on women and children, little attention has been paid to understanding the seasonal variation in the consumption patterns of households engaged in agricultural production [17]. Evidence from South African studies corroborates these findings. Despite all the benefits reported in the available literature (for a review of earlier literature see [18, 19]), very little empirical evidence of the impact of agricultural production on food security in South Africa’s rural areas is available [20]. Mchiza et al. [21], reiterate this, stating that there is a dearth of national data regarding the dietary intakes of adult South Africans as there has never been a national study on adults. The available subnational studies with more localised samples have not used consistent approaches and do not always evaluate food security indicators directly [5, 20]. Even less is reported on the direct nutritional impacts of production - most probably due to the lack of baseline data and weak programme/project design. Very little research has been carried out in South Africa regarding comparative food production and consumption patterns among poor rural people in South Africa. Therefore, this study set out to explore the consumption and production patterns of households in some of the country’s poorest rural communities and to determine the contribution of production to household food security as measured by dietary diversity.

A 2017 review of the General Household Survey data by Stats SA [22] showed that the number of people vulnerable to hunger (based on how often adults and children went hungry because there was not enough food in the household) halved between 2002 and 2007, dropping from 29,3% in 2002 (approximately one in three people) to 13,7% in 2007 (roughly one in seven people). Between 2007 and 2011, there was an increase in the number of persons vulnerable to hunger. This period coincided with the global financial crisis. By 2011, the number of persons vulnerable to hunger had returned to close to pre-crisis levels of 13,1%. However, since 2011 progress stalled, remaining at just above 13,0%. In 2016, 13,4% of the population was vulnerable to hunger.

Given the unacceptably high incidence of food insecurity in the country, government has rolled out a plethora of policies, strategies and programmes aimed at subsistence (for home consumption only) and smallholder production (production for home consumption and sale) in South Africa’s rural areas. However, there has been little assessment of the impact of these programmes [23]. The few available studies show that the production of food at the household level seems to have some benefits for households and children. Selepe and Hendriks [24] caution that the improvements in consumption may not be enough to overcome an alarming state of malnutrition or ensure an adequate diet. For example, Maunder and Meaker’s [25] analysis of the 1999 National Food Consumption Survey data showed that children from households that engaged in agriculture had better intakes of several nutrients, including vitamin A, folate, vitamin B6, vitamin C, calcium and iron, than those from households that did not produce food. However, the scale of production, i.e. household, community or commercial production, was not recorded in the survey.

Results from national surveys [26] show that dietary variety is low (consuming food form four or fewer food groups), particularly among households falling into the low Living Standards Measurement groups. Eggs, legumes and vitamin A rich fruit and vegetables were the least consumed. Households in the former homeland areas seem worst affected. Mchiza et al.’s [21] review of dietary surveys of adults in South Africa between 2000 and 2015 found that micronutrient deficiencies are still highly prevalent. The consumption of fruit, vegetables and dairy was general low. In rural areas this was primarily due to a lack of access to these foods.

The 2011 GHS [27] report shows that for both households with adequate access to food and those with inadequate access, poor households without social grants are less likely to engage in agriculture than households with one social grant. No reasons were provided for the trend. One explanation is that social grants could provide access to the means for production (such as inputs).

Evidence from food security studies conducted between 1994 and 2014 in rural South Africa is not decisive regarding the role of agricultural production on food security. Mudzinganyama [28], Selepe and Hendriks [24], Botha et al. [29], Beery et al. [30], Madlala [31], Lunga [32], Faber and Laubscher [33], Ndlovu [34] and Ngidi [35] report that gardens played a positive role in alleviating food security, while Pereira et al. [36], Prinsloo and Pillay [37] found that food gardens failed to play any significant role in food security [5]. Selepe and Hendriks [24], Shisanya and Hendriks [20], Beery et al. [30], Esterhuyse [38], Faber and Laubscher [33] and van Averbeke and Khose [39], report that household and community gardens can contribute to food security, but cannot assure it [5].

Faber and Benade [40], Faber et al. [41]; Faber et al. [42] and Faber et al. [43] report that crop-based interventions focusing on improving vitamin A intake increased the consumption of yellow/orange-flesh and dark-green leafy vegetables (i.e. linked to pro-vitamin A) among children in KwaZulu-Natal. Seasonal variation in the vitamin-A-rich foods consumed (including traditional leafy vegetables to supplement shortages during some times of the year) exposed the need for year-round consumption. Pereira et al. [36], Mudzinganyama [28] and Ngidi [44], reiterate the seasonal constraints of improved consumption from home production.

There is some evidence that production beyond that for home consumption has greater benefits for diet quality. Mjonono et al. [45] and Hendriks and Msaki [46] investigated the impact of production on the food security of households belonging to a commercially engaged farmers’ organisation and a representative sample of control subsistence (producing for own consumption) households in Embo, KwaZulu-Natal, South Africa. Comparisons between producers selling to a formal supply chain, producers starting to engage in commercial production and subsistence producers showed that selling food improved household food security. These findings support earlier evidence from studies in rural South African that agricultural production can lead to beneficial dietary changes only when production goes beyond subsistence requirements [19, 47, 48].

The evidence of the benefits of crop production on dietary diversity across seasons has not been investigated in South Africa. This study set out to compare the consumption patterns across scales of production and season in four of the country’s poorest rural communities to identify practical guidance on how production could improve consumption and dietary quality across seasons. The study offered a rare opportunity for a team of transdisciplinary researchers (from agricultural economics, crop production, human nutrition and public health) and an experienced non-governmental organisation to bring their knowledge and experience together to investigate a pressing problem and find practical solutions to overcome the problem.

## Methods

Using the priority districts from the Integrated Sustainable Rural Development Programme for the Eastern Cape, KwaZulu-Natal and Limpopo [49], and data from the Health Systems Trust’s [50] Deprivation Index for the North West Province, the most deprived municipalities in these provinces were selected as the sites for this study. Jozini (KwaZulu-Natal), Maruleng (Limpopo) and Ratlou (Northwest) were identified. Initially, Port St Johns Local Municipality in the OR Tambo District was selected as the area for the Eastern Cape sample, but the undulating topography and lack of farming settlements required reconsideration of this site. Ingquza Hill was selected as having the next highest poverty rate and a suitable agricultural context for the Eastern Cape site.

A multistage stratified random sampling technique was applied to identify the communities and sample households for the quantitative survey and qualitative assessments. Enumeration area unit (EAU) orthophoto maps were obtained from Statistics South Africa [51]. All EAUs classified as ‘traditional residential’ for each district were listed. Random computer-generated numbers were used to select two EAUs per local municipality. Sample households were drawn using random computer-generated numbers from the total number of homesteads in each EAU (Table 1).

**Table 1:** Number of households surveyed per community

Site	Location	Summer		Winter	
		Number	Proportion of sample (%)	Number	Proportion of sample (%)
Ingquza Hill	Dubana	21	7.6	29	10.4
	KwaThahle	34	12.2	41	14.6
Jozini	Irrigation Scheme	72	25.9	40	14.3
	KwaJobe	46	16.5	44	15.7
Maruleng	Bochabelo	11	4.0	30	10.7
	Sedawa	25	9.0	30	10.7
Ratlou	Madibogo	47	16.9	49	17.5
	Phitshane	22	7.9	17	6.1
**Total**		278	100.0	280	100.0

Where the EAUs were the sampling frame base (for the sites in Ingquza Hill, Maruleng and Ratlou and one site in Jozini), a list of at least 100 random household numbers were generated and the households were identified and approached in the order of the random sampling list. To be included in the survey, a household had to have at least one child aged between 24 and 59 months with a caregiver present in the homestead and who was willing to participate in the study. Where a household was unavailable or did not meet the criteria for inclusion, the next household on the list was approached until at least 50 households per site were interviewed for the first round of data collection or as many households with small children had been included. Contacting the households for the second round of data collection proved tricky, leading to natural attrition in the sample size.

In the case of KwaZulu-Natal, one site was selected as per the other sites, but a second included farmers from an irrigation scheme (called Makhatini Block 6B or Mjindi). In the case of the irrigation scheme, a list of all farmers belonging to the scheme was obtained (407 members) and the households residing in Jozini (89 members) were identified. Random computer-generated numbers were used to identify a sample of 50 households. A replacement number list was drawn where farmers could not be located, were unavailable for interviews or unwilling to participate in the survey. Due to the process of substitution of additional randomly selected members, all 69 available qualifying households were interviewed from the members of the irrigation scheme (Table 1). The University of Pretoria’s Faculty of Natural and Agricultural Sciences Ethics Committee granted ethical approval for the study (approval number EC130628–066). Qualitative and quantitative data were collected between 2013 and 2015 through focus group discussions (FGDs), key informant interviews and the two-round panel survey to cover both the summer and winter seasons at each site. The data from the survey was cleaned, checked and analysed using Microsoft Excel and SPSS Version 23 [52].

The panel surveys were conducted at each site – one in the drier and less agriculturally productive winter months and one in the summer months. A survey questionnaire was developed by the research team (see Additional file 1). The survey captured information about household crop production, food consumption and household dietary diversity determined through a 24-h recall and calculated using Kennedy et al’s [53]. dietary diversity index as a measure of dietary quality.

Crop-producing households were considered to be those engaged in some form of crop production, such as vegetables, fruit or industrial crops (in this case this included cotton and maize). Data were disaggregated into large-scale farming, community gardens (smaller plots on a shared commonage), school gardens where groups farm smaller plots on a larger commonage, and home gardens. Livestock production was not considered. Non-cropping households did not engage in cropping of any kind, but may have been involved in livestock production. Irrigating households were those engaged in cropping who used some form of irrigation (from buckets to irrigation scheme canals).

## Results

**Fig. 1:**
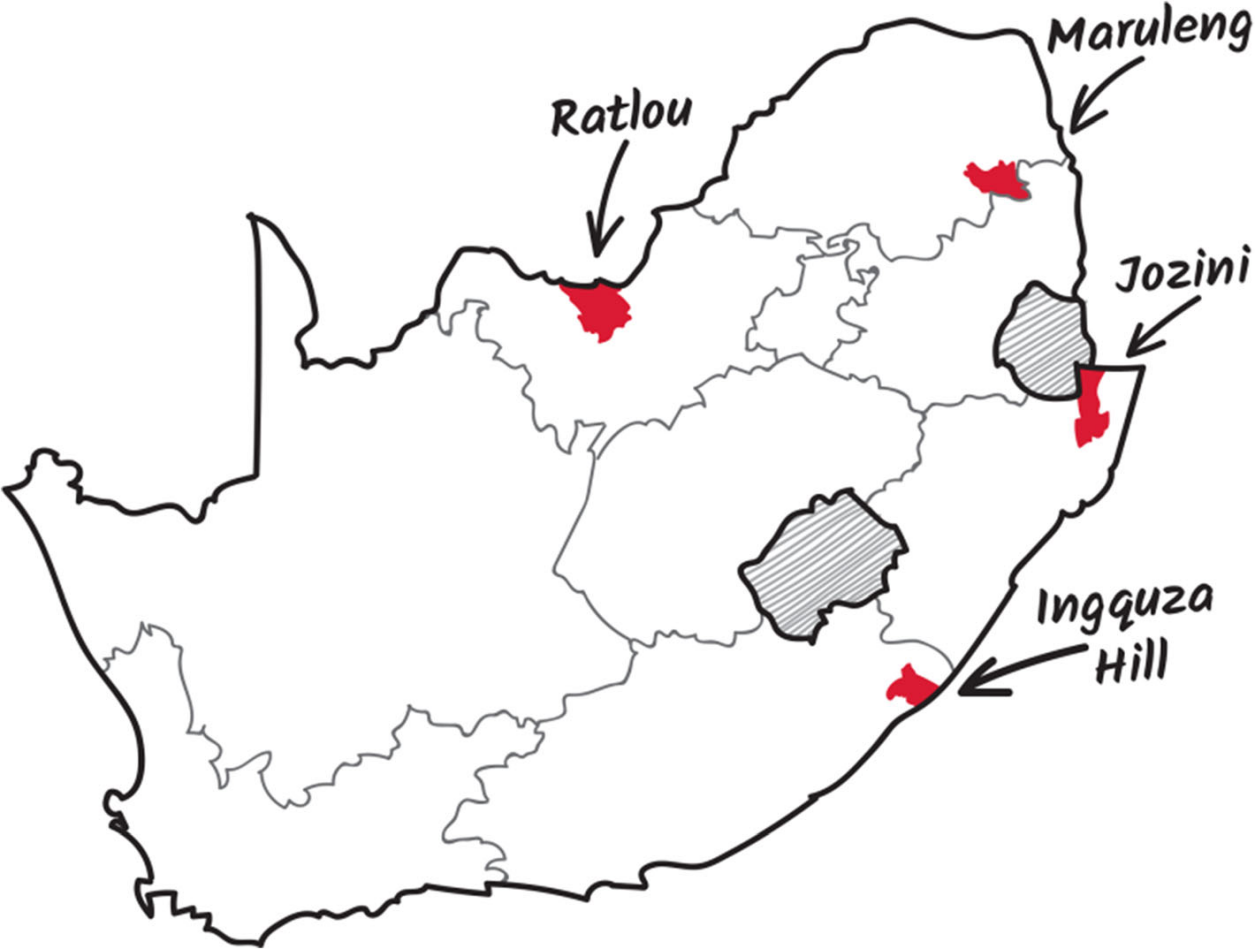
Location of the research sites (source: the authors)

The fieldwork included both qualitative and quantitative assessment and provided rich insight into the daily lives of the communities included in the study. Figure 1 illustrates the location of the sites. The sites were significantly difference in terms of agronomy and hydrology. Table 2 presents a summary of the locations in this regard. The last two rows of the Table indicate that the time of the surveys, the rainfall at each site was within the range of the average rainfall for the location.

**Table 2:** Summary of agronomic and hydrological conditions

Province	KwaZulu-Natal	Eastern Cape	Limpopo	North West
**District**	uMkhanyakude	OR Tambo	Mopani District	Ngaka Modiri Molema
**Local municipality**	Jozini	Ingquza Hill	Maruleng Local Municipality	Ratlou
**Agronomy**	Tropical Ideal weather conditions for agricultural production. In some areas crops can grow year-round – two to three crop cycles a year are possible [54]	Coastal (mixed biome) Ingquza Hill is home to dune forests, swamp forests and coastal forests. Forests are used by local communities and receive little protection due to a lack of formal control. Subsistence agriculture is predominant [59]	Lowveld High agricultural potential with production of tropical and citrus fruit [60].	Grassland/semi-arid This is a semi-arid area with water scarcity.
**Hydrology**	Jozini Dam is a major source of drinking water for people, animals, and irrigation [54].	The area has one large river, the Umzimvubu River, two medium-sized rivers and a number of smaller coastal rivers with limited catchment areas that stretch 60 km inland. The area receives above 800 mm of rainfall a year [55].	Located on the banks of the Blyde River [60] A large population of communal farmers is settled in an area between Hoedspruit and Tzaneen. Seven medium-sized irrigation schemes have been developed in the area, but only two remain functional.	The community is highly dependent on scarce ground water. With the existence of two river systems, one to the north and one in the centre of the area, water tables are relatively low. Borehole water is available, especially in close proximity to the river systems. Agricultural activities should also be located close to water sources [56]. There used to be a dam at Mabule, but, due to floods, it has burst its wall, resulting in the lack of a secure water supply for the villagers.
**Average rainfall** [57, 58]	569 mm	874 mm (Lusikisiki)	566 mm (Hoedspruit)	425 mm (Mabule)
**Annual rainfall for round 1** [57, 58]	August 2012 – July 2013 (October 2013)	August 2012 – July 2013 (July 2013)	August 2013 – July 2014 (November 2014)	August 2013 – July 2014 (November 2014)
	1132 mm	1246 mm	925 mm	593 mm
**Annual rainfall for round 2** [57, 58]	August 2013–July 2014 (July 2014)	August 2013 – July 2014 (October/November 2014)	August 2014 – July 2015 (May 2015)	August 2014 – July 2015 (June 2015)
	924 mm	898 mm	520 mm	411 mm

The communities were located long distances from urban centres. While numerous small retail outlets and informal retailers (*spazas*) were available in the communities, households reported traveling to the nearest urban centre monthly to purchase food in bulk. During the consumption focus groups, several respondents explained that their social grants just cover the costs of the bulk monthly staples (maize meal, white flour, oil and sugar) and the minibus taxi fare to the nearest urban centre. On social grant pay-out day a proliferation of informal traders selling live chickens, eggs, freshly-butchered beef, mutton and offal, as well as fresh produce was observed in the towns.

One of the striking features of the landscape of the Ingquza Hill sites (OR Tambo District) was the vast tracts of rain-fed, terraced farmland that were formerly planted with maize and other staples, but are now in disuse. Subsistence farmers worked on small, fenced home gardens, producing vegetables and keeping a few items of small livestock. A few households still use traditional ox-drawn ploughs and sledges, but these are rare and are only used by a few enthusiastic and dedicated older people who work without much support.

Most of the surveyed households that were engaged in larger-scale crop production were in Jozini (uMkhanyakude District). No households from Ingquza Hill and Ratlou (Ngaka Modiri Molema District) were engaged in larger-scale farming (more than half a hectare). The communities surveyed in Jozini drew water from the Jozini Dam. Despite the dam, FGD discussions focussed on the prevailing drought that significant affected rain-fed home gardening. Irrigation enables year-round production in cooperative gardens and the irrigation scheme. The participants reported that anyone with irrigation equipment could draw water from the river. The irrigation scheme, Mjindi Farming, makes the decisions about water scheme management, as it manages the scheme in Makatini.

At the Maruleng site (Mopani District), agriculture appeared to play a very central role in livelihoods, perhaps more so than in the other study sites, and there seems to be more diverse and vibrant involvement in household, subsistence and smallholder production (beyond production only for household consumption), as well as a greater variety of crops and more involved local management and innovation. Overall, Maruleng producers farmed larger plots and produced more staples than the Eastern Cape and KwaZulu-Natal communities. Some households farmed maize on plots of up to six hectares. Contrary to the other sites, they reportedly produced enough maize to feed their families for the entire year without the need to purchase maize. The Mopani District has a number of small, community-managed irrigation schemes fed by mountain springs. One of these is in Maruleng near the Madeira community.

The Phitshane and Madibogo communities in the Ratlou Municipality, although 80 km apart, share the same typical arid, bushveld savannah-type landscape. With the exception of a few large farms, limited crop production was observed. The lack of engagement in agriculture was attributed to the low rainfall in recent years and the scarcity of water as nearby rivers had dried up and only a few households had access to boreholes. Many households in Phitshane did not have access to piped water in their homes and the communal taps in large sections of the village were often without water. Rain-fed and irrigated production.

The number of households engaged in crop production is presented in Table 3. While we recognise that livestock production may well contribute both food and income to rural households in South Africa, this study focussed on crop production only. Close to nine in ten surveyed households in Ingquza Hill and Jozini were engaged in crop production (90%). More than eight out of ten (82%) of the surveyed households in Maruleng were engaged in cropping. Very few households (four) surveyed in Ratlou were engaged in home gardening. All community gardens were irrigated, while 78% of farmland and 75% of school gardens were irrigated. Just less than half (47%) of home gardens were irrigated. Irrigation was taken to mean any application of supplemental irrigation – from overhead sprinklers using pumps, flood irrigation on irrigation schemes, and municipal water from taps or rain tanks (seen at many Ingquza Hill homesteads), to using a hosepipe or watering can with water drawn from rivers, tributaries, springs, wells, boreholes and tanks (Table 3).

**Table 3:** Households involved in crop production and irrigation

Scale of production		Whole sample		Ingquza Hill		Jozini		Maruleng		Ratlou	
		Involved in cropping	If cropping, irrigating crops	Involved in cropping	If cropping, irrigating crops	Involved in cropping	If cropping, irrigating crops	Involved in cropping	If cropping, irrigating crops	Involved in cropping	If cropping, irrigating crops
Engaged in crop production	Sample size	349	228	68	53	141	122	67	49	65	4
	Yes	242	150	61	26	126	113	51	9	4	2
	Proportion (%)	71.2	65.8	89.7	49.1	89.4	92.6	82.1	18.4	6.2	50.0
Farmland	Sample size	242	73	–	–	126	62	50	11	–	–
	Yes	75	57	–	–	64	54	10	3	–	–
	Proportion (%)	31.1	78.1	–	–	50.8	87.1	20.0	27.3	–	–
Home gardens	Sample size	242	112	61	50	126	19	51	39	4	4
	Yes	120	53	56	25	20	18	40	8	4	2
	Proportion (%)	49.6	47.3	91.8	50.0	15.9	94.7	78.4	20.5	100	50.0
School gardens	Sample size	242	4	61	2	126	2	–	–	–	–
	Yes	4	3	2	1	2	2	–	–	–	–
	Proportion (%)	1.7	75	3.3	50.0	1.6	100	–	–	–	–
Communal gardens	Sample size	242	18	61	–	126	18	–	–	–	–
	Yes	20	18	1	–	19	–	–	–	–	–
	Proportion (%)	8.3	100	1.6	–	15.1	100	–	–	–	–

The highest proportion of household gardens was found at Ingquza Hill (92% of the sample in this area). In Maruleng, 78% of gardens were home gardens. Very few households were engaged in the production of school gardens – only two in Ingquza Hill and two in Jozini (Table 3). One household in Ingquza Hill was involved in a community garden. Half of the home gardens in Ingquza Hill were irrigated. Some 19 households in Jozini were involved in community gardens, which drew water for irrigation from the Mjindi Irrigation Scheme at Makhathini. Larger plots (typically over a hectare) were farmed in Jozini (on the irrigation scheme) and mostly under rain-fed conditions in Maruleng.

Maize was reportedly produced at two school gardens in Ingquza Hill. Dry beans, carrots, maize, onions, potatoes, pumpkin, Swiss chard and sugar beans were produced in home gardens in Ingquza Hill. Far more varieties of crops were produced in Jozini and Maruleng than at the other two sites.

A very high proportion of households in Jozini irrigated their crops. This was primarily due to the availability of abundant water from the dam and a relatively high rainfall compared to the other sites. Yet still, households in Jozini complained of a lack of accessible water. The burden of accessing water in Ingquza Hill and Jozini was a major constraint to crop production. Focus group respondents in Jozini indicated that the irrigation scheme allowed for extended planting times for those participating in the scheme, with reduced water available for non-members.

Production in Maruleng was predominantly rain fed. Some 10% of home gardens in Ingquza Hill were watered with buckets from water tanks (rain harvested). While in Jozini, community and home gardens were typically irrigated with buckets of water drawn from the dam, its tributaries and rivers. Water for the community and school gardens in Jozini was sourced from the irrigation scheme. Very few households mentioned using treadle pumps, sprinklers and municipal water. Canal and flood irrigation was used in the irrigation scheme, although respondents in the FGDs indicated that there were problems with the management and allocation of water – particularly conflicting interests between commercial producers of cotton and maize who tended to dominate the management of the irrigation scheme. Water decision-making is clearly a critical issue in the Jozini community. With a participatory management structure in place in the form of the Water Users’ Association and a wide range of stakeholders that should, in principle, include subsistence, small-scale and commercial farmers, household users, tourism and recreational users, industry, as well as the municipality and tribal authorities, the dynamics are likely to be complex. Current decision-making around water does not prioritise nutrition and food security over commercial needs.

The four households with home gardens in Ratlou grew beans, cabbage, green maize and tomatoes. The crops produced in the community, school and home gardens, and on larger plots of farmland, are presented by site in Tables 4, 5 and 6.

**Table 4:** Crops produced in Ingquza Hill

School gardens	Home gardens
Maize	Carrots
	Dry beans
	Maize
	Onions
	Potatoes
	Pumpkin
	Swiss chard
	Sugar beans

**Table 5:** Crops produced in Jozini

Community gardens	School gardens	Home gardens	Farmland	
*Amadumbe* (*taro*)	Cabbage	Bananas	*Amadumbe*	Mango
Beetroot	Dry Beans	Green beans	Bananas	Mealies
Bananas		Beetroot	Beetroot	Naartjies/ tangerines
Cabbage		Cabbage	Butternut	Onions
Carrots		Calabash	Cabbage	Oranges
Green peppers		Dried beans	Calabash	Potatoes
Maize		Garlic	Carrots	Swiss chard
Onions		Green peppers	Cassava	Sugar beans
Potatoes		*Imifino* ^a^	Dry beans	Sugarcane
Swiss chard		Lettuce	Green peppers	Sweet chillies
Sugarcane		Maize	*Imifino*^a^	Sweet potatoes
		Onions	Lemons	Tomatoes
		Potatoes	*Amadumbe*	
		Swiss chard	Bananas	
		Sweet potatoes	Beetroot	
		Tomatoes	Lettuce	
		Potatoes	Maize	

^a^Indigenous leafy vegetables

**Table 6:** Crops produced in Maruleng

Home gardens		Farmland
Bambara/njugo bean (*ditloo marapo*)	Mealies (green maize)	Green beans
Bananas	*Morogo*	Dry beans
Cabbage	*Morulo tree*	Millet (*leotša)*
Cowpeas (*dinawa*) and leaves (*mokopu*)	Papaya/pawpaw	Maize
Dry beans	Pumpkin	*Morula*
Green beans	Sorghum	*Morogo*^*b*^
Maize	Swiss chard	Swiss chard
Mango	Sugar beans	Green beans
*Morôtsê* (*makataan or Citrullus lanatus*)^a^	Tomatoes	
Millet	Watermelon	

^a^Melon

^b^Indigenous leafy vegetables

It should be noted that the maize typically grown by the households surveyed was either consumed as a green vegetable (referred to here as green mealies) or left to dry on the cob for use in traditional dishes for feasts, festivals and traditional ceremonies. The latter is usually ground on a millstone or at communal granaries. Where the term maize is used in this report, it is used to refer to either green mealies or dried maize. The survey did not record production of these two crops separately, but the FGDs indicated that most maize produced in school, home and community gardens is consumed as green mealies. Dried beans refer to red speckled beans that are left in the field to dry on the plant and then harvested.

Focus group participants in all communities reported climate change, which is explained as rainfall arriving earlier or later in the season and general difficulty with predicting weather conditions. They reported that the rain had come so late in some years that the seeds did not germinate. The lack of predictable weather patterns and the late onset of rain is a deterrent to home gardening, but irrigation enables year-round production in cooperative gardens for those who can participate. According to the FGDs, those who do not have access to irrigation are the ones in the community who go hungry.

The seasonality of production was mapped through FGDs. Quite distinct patterns of production and availability of produce were evident. The diversity (or lack of it) of produce is also evident. The stark contrast between the scarcity of available produce from production in a drier area in Jozini (Hlakaniphani) and Ratlou demonstrates the necessity of water availability to improve the year-round production of vegetables. Reliance on rain-fed production constrains which crops can be planted (those that do not require lots of water and regular watering) and is a serious constraint to production in the drier months. An important consideration that surfaced in the FGDs was the physical drudgery of collecting water from water sources (if available). Households interviewed in the Jozini and Ingquza Hill sites raised this as an important constraint to production. In Ingquza Hill, some households paid community entrepreneurs to fetch water for their household and production uses with pick-up trucks.

Some 60% of households that engaged in farmland crop production in Ingquza Hill and Jozini had sold produce in the year prior to the survey. Just over half (54%) of the households that engaged in larger-scale crop production in Maruleng had sold produce in the same period. Almost half of the households that engaged in community garden production in Jozini had sold produce. Four to five times as many households engaged in home crop production in Jozini (45%) had sold produce in the previous year.

The majority of households consumed foods from only four to eight food groups (Table 7). The average household dietary diversity index for summer was 4.6 (standard deviation 2.14) and 5.0 (standard deviation 2.03) in winter (Table 8). All households consumed maize and so ate foods from the cereals food group. For some households, this staple food was the only food consumed. It seems though that households forgot to report the addition of salt to the cooking water in the preparation of maize porridge. Salt would be classified as a condiment in the dietary diversity assessment but as condiments are not included in the calculation of the dietary diversity index, this would not affect the analysis of dietary diversity.

**Table 7:** Number of food groups consumed in the previous 24 h

Sample	Season	Sample size	One to four food groups	Five to seven food groups	Eight or more food groups
Total sample	Summer	262	40.1	27.8	32.1
	Winter	271	40.2	39.9	19.9
Non-cropping	Summer	101	49.5	30.7	19.8
	Winter	81	51.9	40.7	7.4
Cropping	Summer	159	34.6	25.8	39.6
	Winter	187	34.8	39.5	25.7
Irrigating	Summer	95	20.2	25.1	54.7
	Winter	105	23.8	34.3	41.9
Ingquza Hill	Summer	55	58.2	30.9	10.9
	Winter	69	40.6	52.2	7.2
Jozini	Summer	116	13.8	19	67.2
	Winter	82	28.0	29.3	42.7
Maruleng	Summer	36	75.0	25.0	0.0
	Winter	56	44.6	50.0	5.4
Ratlou	Summer	55	54.5	45.5	0.0
	Winter	64	51.6	40.6	7.8

**Table 8:** The Household Dietary Diversity Scores from the 24-h recall

Site	Season	Sample size	Minimum	Maximum	Mean	Standard error	Standard deviation
Total sample	Summer	159	2	14	7.4	0.327	4.122
	Winter	187	1	14	6.0	0.221	3.021
Ingquza Hill	Summer	55	2	12	4.6	0.289	2.146
	Winter	69	2	10	5.0	0.245	2.036
Jozini	Summer	116	2	14	10.2	0.378	4.070
	Winter	82	2	14	7.3	0.401	3.629
Maruleng	Summer	36	3	7	4.1	0.164	0.984
	Winter	56	2	8	4.8	0.208	1.558
Ratlou	Summer	55	1	7	4.2	0.174	1.290
	Winter	64	1	9	4.7	0.215	1.719
Non-cropping	Summer	101	1	14	6.0	0.399	4.014
	Winter	81	2	9	4.7	0.178	1.603
Cropping	Summer	159	2	14	7.4	0.327	4.122
	Winter	187	1	14	6.0	0.221	3.021
Irrigating	Summer	95	2	14	8.9	0.421	4.099
	Winter	105	2	14	7.2	0.325	3.331

Table 9 shows that only three foods groups (cereals, other vegetables, oils and fats) were consumed by more than half of the households in summer, followed by roughly one in three households that included foods from the white roots and tubers, dark green vegetables, and meat and milk products groups in summer. In winter, the consumption of cereals, and oils and fats remained consistent, but the consumption of dark green leafy vegetables and meat dropped considerably. The consumption of dried legumes and milk increased in winter.

**Table 9:** Food group consumption for cropping and non-cropping households from the 24-h recall

	Season	Total sample		Non-cropping		Cropping		Irrigating	
		Number	Proportion	Number	Proportion	Number	Proportion	Number	Proportion
Sample size	Summer	264		103		83		75	
	Winter	279		83		193		108	
Cereals	Summer	255	96.2	99	96.1	83	100	74	98.7
	Winter	273	97.5	83	100	186	96.4	102	94.4
White roots and tubers	Summer	125 (of 265)	47.2	47	45.6	31	37.3	20	26.7
	Winter	113	40.4	31	37.3	81	42.0	58	53.7
Orange-fleshed vegetables	Summer	83	31.6	23 (of 102)	22.5	15	18.1	10	13.3
	Winter	57	20.4	15	18.1	42	21.8	31	28.7
Dark green leafy vegetables	Summer	132	50.2	39 (of 102)	38.2	20	24.1	16	21.3
	Winter	93	33.2	20	24.1	73	37.8	55	50.9
Other vegetables	Summer	196	74.2	61 (of 102)	59.8	37	44.6	45 (of 72)	62.5
	Winter	180 of 274)	65.7	37	44.6	140	72.5	89 (of 106)	84.0
Orange-coloured fruit	Summer	72	27.3	24 (of 102)	23.5	4	4.8	5 (of 74)	6.8
	Winter	35 (of 277)	12.6	4	9.6	31 (of 191)	16.2	26 (of 107)	24.3
Other fruit	Summer	100	37.9	24 (of 102)	23.5	8	9.6	16	21.3
	Winter	69	24.7	8	9.6	60	31.1	43	39.8
Organ meat	Summer	79 (of 263)	30.0	28 (of 102)	27.7	13 (of 82)	15.9	5	6.7
	Winter	32 (of 278)	11.5	13 (of 82)	15.9	19	31.1	14	13.0
Meat	Summer	140	53.0	47 (of 102)	46.1	46	55.4	28	37.3
	Winter	144	51.6	46	55.4	97	50.3	67	62.0
Eggs	Summer	76	28.8	25 (of 102)	24.5	4	4.8	4	5.3
	Winter	39	13.9	4	4.8	35	18.1	29	26.9
Fish and seafood	Summer	98 (of 263)	37.3	35 (of 101)	34.7	12 (of 82)	14.6	9	12.0
	Winter	65	23.4	12 (of 82)	4.8	53	27.5	44	40.7
Dried beans and legumes	Summer	96	36.4	25 (of 102)	24.5	8	9.6	16	21.3
	Winter	79	28.2	8	9.6	71	36.8	54	50.0
Milk and milk products	Summer	136	51.1	51 (of 102)	50.0	35	42.2	29	38.7
	Winter	124	44.4	35	42.2	88	45.6	55	50.9
Oils and fats	Summer	238 (of 265)	89.9	91	90.4	73	88.0	59	78.7
	Winter	243	87.1	73	88.0	167	86.5	99	91.7

Of concern was that only 32% of the households surveyed had consumed food from eight or more food groups in the previous day in summer, while 20% of the households had consumed food from these food groups in the previous day in winter. In summer, 56% of households included other vegetables (mostly tomatoes, onions, green peppers and wild/indigenous vegetables) in their meals, while 61% did so in winter. Households also consumed white roots and tubers (44% in summer and 43% in winter), dark green leafy vegetables (29% in summer and 15% in winter) and orange-fleshed vegetables (22% in summer and 24% in winter). However, these were not consumed every day or in large quantities.

Engagement in production influenced dietary diversity. Far more cropping households consumed foods from eight or more food groups in both summer and winter (Table 9). The data presented in Table 9 show that 40% of households involved in cropping consumed foods from eight or more food groups in summer and 26% did so in winter. This was remarkably different to the non-cropping households, where only 20% consumed foods from eight or more food groups in summer and 7% did so in winter. Over half (55%) of irrigating households consumed foods from eight or more food groups in summer and 42% consumed these foods in winter. Even though most foods were purchased, cropping increased the availability of foods for home consumption.

The analysis does not indicate a strong influence of crop production on the consumption of fruit and vegetables in summer (Table 9). However, proportionally fewer cropping households consumed orange-fleshed vegetables in summer, but consumed more than non-cropping households consume in winter. The same pattern was seen for the consumption of dark green leafy vegetables, other vegetables, orange-coloured fruit, other fruit, as well as dried beans and legumes. This shows a more positive influence of cropping on the consumption of fruit and vegetables in winter than in summer. This result was not expected, as the number of crops that can produce edible portions in winter is rather limited. Some crops such as beans are produced in summer and preserved (dried) for consumption in winter. The cultural preference is for dried beans rather than fresh green beans. Crops such as pumpkin and butternut are also stored for consumption later. Another explanation may be that savings from consumption in summer were used to purchasing these foods in winter, post-harvest. This was certainly true for households engaged in farmland cultivation (larger scale production) where consumption patterns improved in winter, post-harvesting of the main crops. However, farmland cultivation was only carried out under irrigated conditions.

The dietary diversity of non-crop-producing households was lower than that of crop-producing households in both winter and summer (Table 8), and decreased by at least one food group in winter. Households engaged in cropping had higher average HDDSs. Irrigation increased the average HDDS of crop-producing households even further. The HDDS for irrigating households increased from an average of 7.1 food groups in summer to 8.9 in winter; probably due to the availability of income form the previous season that enabled the purchasing of more diverse foods in winter.

Cropping was significantly and positively correlated with the consumption of orange-fleshed vegetables, dark green leafy vegetables, other vegetables, other fruit and dried beans and legumes (Table 10). Irrigating and farmland (larger-scale) production were positively correlated to the consumption of other vegetables, other fruit, and dried beans and legumes.

**Table 10:** Correlations (Spearman’s) of food group consumption and scale of farming

Food group		Cropping	Irrigating	Farm- land	Home garden	School garden	Community garden
White roots and tubers	Correlation coefficient	0.027	0.073	0.084	−0.138^a^	0.031	0.063
	Significance (two-tailed)	0.531	0.103	0.051	0.001	0.470	0.140
	Sample size	539	506	543	545	545	545
Orange-fleshed vegetables	Correlation coefficient	0.088^b^	−0.027	0.083	−0.083	0.044	0.041
	Significance (two-tailed)	0.040	0.542	0.054	0.054	0.301	0.335
	Sample size	537	504	540	542	542	542
Dark green leafy vegetables	Correlation coefficient	0.142^a^	−0.059	0.153^a^	−0.194^a^	0.003	0.156^a^
	Significance (two-tailed)	0.001	0.187	0.000	0.000	0.942	0.000
	Sample size	537	504	540	542	542	542
Other vegetables	Correlation coefficient	0.264^a^	−0.234^a^	0.285^a^	−0.075	− 0.068	0.142^a^
	Significance (two-tailed)	0.000	0.000	0.000	0.082	0.117	0.001
	Sample size	533	500	536	538	538	538
Orange-coloured fruit	Correlation coefficient	0.085	−0.034	0.138^a^	−0.172^a^	0.025	0.082
	Significance (two-tailed)	0.050	0.447	0.001	0.000	0.557	0.055
	Sample size	536	503	539	541	541	541
Other fruit	Correlation coefficient	0.212^a^	−0.121^a^	0.167^a^	−0.085^b^	0.029	0.081
	Significance (two-tailed)	0.000	0.007	0.000	0.049	0.501	0.059
	Sample size	538	505	541	543	543	543
Dry beans and legumes	Correlation coefficient	0.222^a^	−0.150^a^	0.134^a^	−0.075	−0.009	0.144^a^
	Significance (two-tailed)	0.000	0.001	0.002	0.079	0.835	0.001
	Sample size	538	505	541	543	543	543

^a^Correlation is significant at the 0.01 level (two-tailed)

^b^Correlation is significant at the 0.05 level (two-tailed)

## Discussion

Income from farmland production and irrigated agriculture led to increased intakes of fruit and vegetables in general, but also meat, eggs, fish, milk, roots and tubers. This shows that there is potential for greater improvement in dietary diversity and quality if households scale up production to produce enough food to sell.

Home gardening led to a significant positive increase in the consumption of white roots and tubers, dark green leafy vegetables, orange-coloured fruit and other fruit in the 24 h prior to the survey (Table 10). Participation in a community garden led to significant increases in the consumption of dark green leafy vegetables and other vegetables. School gardening did not demonstrate any statistical relationships with the consumption of foods from the crop-related food groups.

The study found an encouraging link between engaging in agriculture and diet quality. Engagement in crop production increased the availability of vegetables and, in some cases, fruit (when in season). This improved households’ dietary diversity. Income from farmland production and irrigated agriculture led to increased intakes of fruit and vegetables in general, but also of meat, eggs, fish, milk, roots and tubers. However, the scale of production and year-round availability was a constraint to accessing an adequately diversified diet.

Yet, without marketing opportunities at both at the community and food system level, there are few incentives to increase the production of these crops beyond home consumption. Yet, our findings show that producing beyond that solely for home consumption has greater benefits for dietary diversity and a consumption-smoothing effect during the post-harvest period. It is therefore, essential that policies and programmes pay attention to the development of market infrastructure and linkages to markets for the uptake of produce.

From the findings, we are able to recommend that production and education programmes focus on s*trengthen* existing good consumption patterns and *promoting* the consumption of foods that can improve dietary diversity. Table 11 presents a list of potentially important crops for each area that could improve food consumption, based on the eating and purchasing patterns of the communities investigated.

**Table 11:** Recommendations to improve dietary intake

Food Group	Specific crops (alphabetically)	Recommendations			
		Ingquza Hill	Jozini	Maruleng	Ratlou
Dark green leafy vegetables	Beetroot*	Promote and strengthen existing good patterns	Strengthen existing good patterns	Promote existing good patterns	Promote existing good patterns
	Legumes*				
	Pumpkins*				
	Spinach				
	Sweet potatoes*				
	African leafy vegetables (‘wild’ or cultivated)				
Other vegetables	Beetroot	Promote and strengthen existing good patterns	Strengthen existing good patterns	Strengthen existing good patterns	Promote existing good patterns
	Cabbage				
	Cucumber Eggplant (brinjal)# Green beans Gem squash/‘Calabash’/other squash and pumpkin Green peppers^&^ Lettuce Onions^&^ Tomatoes Zucchini (baby marrow)#				
Other fruit	Apples	Promote existing good patterns	Promote and strengthen existing good patterns	Promote and strengthen existing good patterns	Promote existing good patterns
	Avocados				
	Bananas				
	Berries				
	Citrus fruit				
	Figs				
	Guava (this tree has been classified as an invader species, and although high in nutrition, should not be recommended for cultivation)				
	Pears				
	Pineapples				
	Plums#				
	Watermelons				
Short-term: Orange-fleshed vegetables	Carrots Dark orange pumpkin, butternut or squash Orange sweet potatoes	Promote existing good patterns	Strengthen existing good patterns	Promote existing good patterns	Promote existing good patterns
Longer term: Orange-coloured fruit	Apricots	Promote existing good patterns	Promote existing good patterns	Promote existing good patterns	Promote existing good patterns
	Loquats				
	Mangos				
	Papaya				
	Orange peaches				
	Spanspek (cantaloupe)#				

*Refers to the consumption of the leaves of the crops

^&^ Presumably small quantities are eaten; thus no nutrient intake significance (flavour and diversity considerations)

# Acceptability unknown

## Conclusions

This is the first comparative paper between the poorest communities in South Africa. The findings show that, contrary to popular opinion and rhetoric, a significant number of households in South Africa’s poorest rural communities were engaged in production at some level, supplementing their diets in the areas where crop production was possible (Ingquza Hill, Jozini and Maruleng). Very few households engaged in agriculture in Ratlou due to the aridity of the area.

Rather than perpetuating the rhetoric of deagrarianisation and claiming that social grants are a disincentive to production in South Africa’s rural areas, it is essential for politicians and the science community alike to recognise the role that household and small-scale crop production plays in supporting household consumption and the provision of essential micronutrients. The study shows that people continue to produce food in these communities despite the constraints and disincentives. Crop production, along with educational programmes to promote the diversification of diets should be supported in South Africa’s rural communities. Combined with social grants that help purchase basic food staples and other non-food goods and services, production can fill nutrient gaps, improving dietary quality and offering opportunities for incomes when scaled beyond production for home consumption.

The findings call for more carefully designed production support programmes that go beyond a focus on maize production to cover the production of fruit and vegetables in a variety of production systems (from rainfed production to innovative and water-saving techniques such as vertical gardens). Support should include the provision of basic water harvesting and irrigation infrastructure, greater access to quality inputs and appropriate extension support.

Research is needed to overcome the seasonality constraints including the development of early- and late-maturing crops to extend the growing season and make food from own production available for longer periods. The development and testing of technologies and practices that are appropriate to home and small scale production conditions (including pest and disease management) should be prioritised by researchers, government programmes and extension agents. Water harvesting practices and systems are essential to enable food production in more homes and the availability of water for irrigation in winter. The provision of boreholes and piped water is essential in drier areas such as Ratlou.

## Supplementary information

**Additional file 1.** Survey Questionnaire

## Data Availability

The data that support the findings of this study are available from the corresponding author, Sheryl Hendriks, upon reasonable request. The data are not publicly available due to their containing information that could compromise the privacy of research participants.

## Abbreviations

DFID: Department for International Development
EAU: Enumerator Area Unit
FGD: Focus Group Discussion
GHS: General Household Survey
HDDS: Household dietary diversity index
MDG: Millennium Development Goal
NGO: Non-government Organisation
SANHANES-1: South African National Health and Nutrition Examination Survey
SPSS: Statistical Package for Social Sciences
StatsSA: Statistics South Africa
USAID IYCN: United States Agency for International Development’s Infant and Young Child Nutrition Programme
WRC: Water Research Commission of South Africa
