# Supplementary material for: Considerations for the design of nutrition-sensitive production programmes in rural South Africa
Source: BMC Public Health. 2020 Sep 10;20:1383. doi: 10.1186/s12889-020-09445-3 (PMC7488396; doi:10.1186/s12889-020-09445-3)
Supplement: Supplementary file 1 — Additional file 1. Survey Questionnaire [file 12889_2020_9445_MOESM1_ESM.docx]

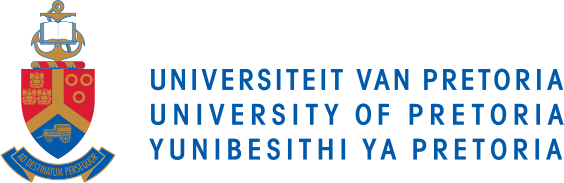


**Project name: Current rain-fed and irrigated production of food crops and its potential to meet all year round nutritional requirements of rural poor people in North West, Limpopo, KwaZulu-Natal and Eastern Cape Provinces**

**A: Particulars of the dwelling**

A1: PSU Number_____

A2: Questionnaire number of this household: ­­­­____

A3: Telephone number of enumerated household: _ _ _ _ _ _ _ _ _ _

A4: Total number of persons in household:

Survey Officer name:

Date of survey: __/_ _/_ ___

*The following information must be obtained for every person who is considered a member of the household.*  *Only add persons who had stayed here for at least four nights on average per week for the last four weeks.* ***Do not forget babies.***

*If there are more than 10 persons in the household, use a second questionnaire.*

|  |  | 01 | | | | 02 | | | | 03 | | | | 04 | | | | 05 | | | | 06 | | | | 07 | | | | 08 | | | | 09 | | | | 10 | | | |
| --- | --- | --- | --- | --- | --- | --- | --- | --- | --- | --- | --- | --- | --- | --- | --- | --- | --- | --- | --- | --- | --- | --- | --- | --- | --- | --- | --- | --- | --- | --- | --- | --- | --- | --- | --- | --- | --- | --- | --- | --- | --- |
| **A** | **First name and surname *First name:*** *Write down first name and surname of each member of the household, starting with the head or acting head. If more than one head or acting head take the oldest.*  ***Surname:*** |  | | | |  | | | |  | | | |  | | | |  | | | |  | | | |  | | | |  | | | |  | | | |  | | | |
|  |  |  |  |  |  |  |  |  |  |  |  |  |  |  |  |  |  |  |  |  |  |  |  |  |  |  |  |  |  |  |  |  |  |  |  |  |  |  |  |  |  |
|  |  |  |  |  |  |  |  |  |  |  |  |  |  |  |  |  |  |  |  |  |  |  |  |  |  |  |  |  |  |  |  |  |  |  |  |  |  |  |  |  |  |
|  |  |  | | | |  | | | |  | | | |  | | | |  | | | |  | | | |  | | | |  | | | |  | | | |  | | | |
|  |  |  | | | |  | | | |  | | | |  | | | |  | | | |  | | | |  | | | |  | | | |  | | | |  | | | |
|  |  |  |  |  |  |  |  |  |  |  |  |  |  |  |  |  |  |  |  |  |  |  |  |  |  |  |  |  |  |  |  |  |  |  |  |  |  |  |  |  |  |
|  |  |  |  |  |  |  |  |  |  |  |  |  |  |  |  |  |  |  |  |  |  |  |  |  |  |  |  |  |  |  |  |  |  |  |  |  |  |  |  |  |  |
|  |  |  | | | |  | | | |  | | | |  | | | |  | | | |  | | | |  | | | |  | | | |  | | | |  | | | |
| **B** | Has ……….... stayed here (in this household) for at least four nights on average per week during the last four weeks?  1 = Yes  2 = No ***If “No”, End of interview*** | 1  2 | | | | 1  2 | | | | 1  2 | | | | 1  2 | | | | 1  2 | | | | 1  2 | | | | 1  2 | | | | 1  2 | | | | 1  2 | | | | 1  2 | | | |
| **C** | **Is ... a male or a female?**  1 = Male  2 = Female | 1  2 | | | | 1  2 | | | | 1  2 | | | | 1  2 | | | | 1  2 | | | | 1  2 | | | | 1  2 | | | | 1  2 | | | | 1  2 | | | | 1  2 | | | |
| **D** | **What is …?’s date of birth and age in completed years?**  **Day of Birth:**  *Example of day 05*  **Month of birth:**  *Example of month 11*  **Year of birth:**  *Example of year 2007*  **Age in years**  *Less than one year = 0* | d d m m  y y y y | | | | d d m m  y y y y | | | | d d m m  y y y y | | | | d d m m  y y y y | | | | d d m m  y y y y | | | | d d m m  y y y y | | | | d d m m  y y y y | | | | d d m m  y y y y | | | | d d m m  y y y y | | | | d d m m  y y y y | | | |
|  |  |  |  |  |  |  |  |  |  |  |  |  |  |  |  |  |  |  |  |  |  |  |  |  |  |  |  |  |  |  |  |  |  |  |  |  |  |  |  |  |  |
|  |  |  | | | |  | | | |  | | | |  | | | |  | | | |  | | | |  | | | |  | | | |  | | | |  | | | |

**SECTION 1: HOUSEHOLD SPECIFIC CHARACTERISTICS**

***This section covers particulars of each person in the household***

**Go to Q 1.3a**

|  |  | 01 | 02 | 03 | 04 | 05 | 06 | 07 | 08 | 09 | 10 |
| --- | --- | --- | --- | --- | --- | --- | --- | --- | --- | --- | --- |
| **1.1** | **What is ……’s relationship to the head of the**  **household? (i.e. to the person in column 1)**  1 = Head/acting head  2 = Husband/wife/partner of person 01  3 = Son/daughter/stepchild/adopted child of  person 01  4 = Brother/sister/stepbrother/stepsister of  person 01  5 = Father/mother/stepfather/stepmother of  person 01  6 = Grandparent/great grandparent of person 01  7 = Grandchild/great grandchild of person 01  8 = Other relative (e.g. in-laws or aunt/uncle)  of person 01  9 = Non-related persons |  |  |  |  |  |  |  |  |  |  |
| **1.2** | **What is ……’s present marital status?**  1 = Legally married  2 = Living together like husband and wife  3 = Divorced  4 = Separated, but still legally married  5 = Widowed  6 = Single, but have been living together with  someone as husband/wife before  7 = Single and have never been married/never  lived together as husband/wife before |  |  |  |  |  |  |  |  |  |  |
| **1.3** | **Does ….’s spouse/partner live in this**  **household?**  1 = Yes  2 = No ***Go to Q1.3a*** | 1  2 | 1  2 | 1  2 | 1  2 | 1  2 | 1  2 | 1  2 | 1  2 | 1  2 | 1  2 |

**EDUCATION**

***Ask for all household members. Read out: Now I am going to ask you questions related to education for each member of the household***

|  |  | 01 | 02 | 03 | 04 | 05 | 06 | 07 | 08 | 09 | 10 |
| --- | --- | --- | --- | --- | --- | --- | --- | --- | --- | --- | --- |
| **1.4** | **What is the highest level of education that**  **…… has successfully completed?** *Diplomas or certificates must be of six months plus study duration full-time (or equivalent) to be included*  98 = No schooling  00 = Grade R/0  01 = Grade 1/ Sub A/Class 1  02 = Grade 2 / Sub B/Class 2  03 = Grade 3/Standard 1/ ABET 1(Kha Ri Gude, Sanli)  04 = Grade 4/ Standard 2  05 = Grade 5/ Standard 3/ ABET 2  06 = Grade 6/Standard 4  07 = Grade 7/Standard 5/ ABET 3  08 = Grade 8/Standard 6/Form 1  09 = Grade 9/Standard 7/Form 2/ ABET 4  10 = Grade 10/ Standard 8/ Form 3  11 = Grade 11/ Standard 9/ Form 4  12 = Grade 12/Standard 10/Form 5/Matric  (No Exemption)  13 = Grade 12/Standard 10/Form 5/Matric (Exemption *)  14 = matric plus Diploma or degree |  |  |  |  |  |  |  |  |  |  |
|  |  |  |  |  |  |  |  |  |  |  |  |
|  |  |  |  |  |  |  |  |  |  |  |  |

***Ask for all household members aged 0-5 years.***

|  |  | 01 | 02 | 03 | 04 | 05 | 06 | 07 | 08 | 09 | 10 |
| --- | --- | --- | --- | --- | --- | --- | --- | --- | --- | --- | --- |
| **1.5** | **Which of the following does the child**  **currently attend?**  1 = Grade R  2 = Pre-school / nursery school/  Grade 00/Grade 000  3 = Creche / educare centre  4 = Day-mother – out of home care  5 = None  6 = Do not know |  |  |  |  |  |  |  |  |  |  |
|  |  |  |  |  |  |  |  |  |  |  |  |
|  |  |  |  |  |  |  |  |  |  |  |  |

|  |  | 01 | 02 | 03 | 04 | 05 | 06 | 07 | 08 | 09 | 10 |
| --- | --- | --- | --- | --- | --- | --- | --- | --- | --- | --- | --- |
| **1.6** | **Does…. attend a school where food is given to the children?**  1 = Yes  2 = No ***Go to Q1.23a***  3 = Do not know ***Go to Q1.23a*** | 1  2  3 | 1  2  3 | 1  2  3 | 1  2  3 | 1  2  3 | 1  2  3 | 1  2  3 | 1  2  3 | 1  2  3 | 1  2  3 |

|  |  | 01 | 02 | 03 | 04 | 05 | 06 | 07 | 08 | 09 | 10 |
| --- | --- | --- | --- | --- | --- | --- | --- | --- | --- | --- | --- |
| **1.7** | **On how many days in a week does …. eat the food provided at school?** *If yes, specify how regularly food is eaten.*  1 = number of times per week  2 = Do not know |  |  |  |  |  |  |  |  |  |  |

**SECTION 2: ECONOMIC ACTIVITY STATUS**

***Ask for all household members. Read out: Now I am going to ask you questions related to occupation for each member of the household***

|  |  | 01 | 02 | 03 | 04 | 05 | 06 | 07 | 08 | 09 | 10 |
| --- | --- | --- | --- | --- | --- | --- | --- | --- | --- | --- | --- |
| **2.1** | **What is your Economic activity Status?**  01=Subsistence farmers  02=Commercial farmers  03=Parastatal employees  04=Formal sector private employees,  05=Self-employed outside agriculture,  06=Unpaid family worker  07=Workers not elsewhere classified, based on employment status  08=Unemployed,  09=Inactive, those whose main current activity was not working or running a business |  |  |  |  |  |  |  |  |  |  |
|  |  |  |  |  |  |  |  |  |  |  |  |

**ECONOMIC ACTIVITIES**

***Ask for all household members 15 years and older***

|  |  |  | 01 | 02 | 03 | 04 | 05 | 06 | 07 | 08 | 09 | 10 |  |
| --- | --- | --- | --- | --- | --- | --- | --- | --- | --- | --- | --- | --- | --- |
|  | **2.2** | **During the last calendar week (Sunday to Saturday) did ….. work for a wage, salary, commission or any payment in kind (including paid domestic work), even if it was for only one hour?** *Examples: a regular job, contract, casual or piece work for pay, work in*  *exchange for food or housing, paid domestic work.*  1 = Yes  2 = No  3 = Do not know | 1  2  3 | 1  2  3 | 1  2  3 | 1  2  3 | 1  2  3 | 1  2  3 | 1  2  3 | 1  2  3 | 1  2  3 | 1  2  3 |  |
|  | **2.3** | **During the last calendar week (Sunday to Saturday) did … run or do any kind of business, big or small, for yourself or with one or more partners, even**  **if it was for only one hour?** *Examples: Commercial farming, selling things, making things for sale, construction, repairs, guarding cars, brewing beer, collecting wood or water for sale, hairdressing, etc.*  1 = Yes  2 = No  3 = Do not know | 1  2  3 | 1  2  3 | 1  2  3 | 1  2  3 | 1  2  3 | 1  2  3 | 1  2  3 | 1  2  3 | 1  2  3 | 1  2  3 |  |
|  | **2.4** | **During the last calendar week (Sunday to Saturday) did ...… do any work for which they were paid in some way besides cash?** *Examples: Commercial farming, production of agricultural produce to sell, help to sell things, make things for sale or exchange, doing the accounts, cleaning up for the business, etc.*  1 = Yes, ate food on site  2 = Yes, was given a food ration to take home  3 = Yes, was given non-food items  4 = Do not know | 1  2  3 | 1  2  3 | 1  2  3 | 1  2  3 | 1  2  3 | 1  2  3 | 1  2  3 | 1  2  3 | 1  2  3 | 1  2  3 |  |

|  |  | 01 | 02 | 03 | 04 | 05 | 06 | 07 | 08 | 09 | 10 |
| --- | --- | --- | --- | --- | --- | --- | --- | --- | --- | --- | --- |
| **2.5** | **What is ……’s total salary/pay at his/her main job?** *Including overtime, allowances and bonus, before any tax or deductions. Give amount in whole figures, without any text or decimals.* If “NONE”, “REFUSE” **or** “DO NOT KNOW write 999 999 999 |  |  |  |  |  |  |  |  |  |  |
| **2.6** | **Ask only if an amount is given in Q4.4**  **Is this….**  1 = Per week  2 = Per month  3 = Annually | 1  2  3 | 1  2  3 | 1  2  3 | 1  2  3 | 1  2  3 | 1  2  3 | 1  2  3 | 1  2  3 | 1  2  3 | 1  2  3 |
|  | | | | | | | | | | | |

**SECTION 3: HEALTH AND GENERAL FUNCTIONING**

***Ask for all household members. Read out: Now I am going to ask you health-related questions for each member of the household***

|  |  |  | 01 | 02 | 03 | 04 | 05 | 06 | 07 | 08 | 09 | 10 |  |
| --- | --- | --- | --- | --- | --- | --- | --- | --- | --- | --- | --- | --- | --- |
|  | **3.1** | **Has …..been unable to perform their usual duties (housework, employment) for 30 days or more during the past year due to illness?**  *Read all the options*  01 = Yes  02 = No |  |  |  |  |  |  |  |  |  |  |  |
|  |  |  |  |  |  |  |  |  |  |  |  |  |  |
|  |  |  |  |  |  |  |  |  |  |  |  |  |  |

***Read out: I am now going to ask about the general functioning of persons within the household***

**SECTION 4: SOCIAL GRANTS AND SOCIAL RELIEF**

***Ask for all household members***

***Read out: I am now going to ask about the use of social grants and social relief***

|  |  | 01 | 02 | 03 | 04 | 05 | 06 | 07 | 08 | 09 | 10 |
| --- | --- | --- | --- | --- | --- | --- | --- | --- | --- | --- | --- |
| **4.1** | **Does anyone in this household receive a social grant, pension or social relief assistance from the Government?**  1 = Yes  2 = No ***Go to Q3.4***  3 = Do not know ***Go to Q3.4*** | 1  2  3 | 1  2  3 | 1  2  3 | 1  2  3 | 1  2  3 | 1  2  3 | 1  2  3 | 1  2  3 | 1  2  3 | 1  2  3 |
| **4.2** | *If “Yes” in Q3.1a*  **Does … receive a(n).......?** *Answer for each person who qualified for the grant and NOT for the person who applied on behalf of/physically receives the money. Someone who used to work for the Government and receive a pension do* ***not get*** *an old age grant*  *Read all the options*  1 = Old-age grant (60-74;R1200; 75+; R1220)  2 = Disability grant (18-59;R1200)  3 = Child support grant (0-17;R280)  4 = Care dependency grant (0-17;R1200)  5 = Foster child grant (<22; R770)  6 = War veterans grant (60+; R1220)  7 = Grant-in-aid (R250 and should  have another grant)  8 = Social relief of distress | Yes No  1 2  1 2  1 2  1 2  1 2  1 2  1 2  1 2 | Yes No  1 2  1 2  1 2  1 2  1 2  1 2  1 2  1 2 | Yes No  1 2  1 2  1 2  1 2  1 2  1 2  1 2  1 2 | Yes No  1 2  1 2  1 2  1 2  1 2  1 2  1 2  1 2 | Yes No  1 2  1 2  1 2  1 2  1 2  1 2  1 2  1 2 | Yes No  1 2  1 2  1 2  1 2  1 2  1 2  1 2  1 2 | Yes No  1 2  1 2  1 2  1 2  1 2  1 2  1 2  1 2 | Yes No  1 2  1 2  1 2  1 2  1 2  1 2  1 2  1 2 | Yes No  1 2  1 2  1 2  1 2  1 2  1 2  1 2  1 2 | Yes No  1 2  1 2  1 2  1 2  1 2  1 2  1 2  1 2 |
|  | | | | | | | | | | | |

**SECTION 5: ANTHROPOMETRY: the following measurements should be recorded for all children under 5 years of age and their caregivers**

|  |  | 01 | 02 | 03 | 04 | 05 | 06 | 07 | 08 | 09 | 10 |
| --- | --- | --- | --- | --- | --- | --- | --- | --- | --- | --- | --- |
| **5.1** | **Is any female in the household pregnant?**  1 = Yes  2 = No | 1  2  3 | 1  2  3 | 1  2  3 | 1  2  3 | 1  2  3 | 1  2  3 | 1  2  3 | 1  2  3 | 1  2  3 | 1  2  3 |
| **5.2** | **Is the person older than 2 years (24 months) and under 5 years of age (<60 months) and at home or a primary caregiver of that child?**  1 = Yes  2 = No | 1  2 | 1  2 | 1  2 | 1  2 | 1  2 | 1  2 | 1  2 | 1  2 | 1  2 | 1  2 |
|  | Measure weight of (household member name). Measure twice with a digital scale. Measurements are recorded in kilograms with two decimals. | | | | | | | | | | |
|  | Weight measurement 1 |  |  |  |  |  |  |  |  |  |  |
| **5.3** | Weight measurement 2 |  |  |  |  |  |  |  |  |  |  |
|  | **2.** **M**easure height of children who are 2 – 5 years (household member name). Measure twice with stadiometer in centimeter (cm). | | | | | | | | | | |
|  | Height measurement 1 |  |  |  |  |  |  |  |  |  |  |
| **5.4** | Height measurement 2 |  |  |  |  |  |  |  |  |  |  |
| 5.5 | Mid-upper arm circumference (MUAC) for the children under 5: Measure twice with narrow tape in millimeters (mm). Measures (4-5) should only be done to children under the age of 5 (60 months). | | | | | | | | | | |
|  | MUAC Reading 1 (force entry) |  |  |  |  |  |  |  |  |  |  |
|  | MUAC Reading 2 (force entry) |  |  |  |  |  |  |  |  |  |  |
| 5.6 | Last entry on the Road to Health booklet of children under 5 years of age | | | | | | | | | | |
|  | Date of entry |  |  |  |  |  |  |  |  |  |  |
|  | Age of the child in months |  |  |  |  |  |  |  |  |  |  |
|  | Weight of the child in kg, 2 decimal places |  |  |  |  |  |  |  |  |  |  |

**SECTION 6:** **GENERAL HOUSEHOLD INFORMATION AND SERVICE DELIVERY**

*Ask a responsible person in the household to answer on behalf of the household.*

**HOUSING *Ask all households***

| **6.1 Indicate the type of main dwelling that the household occupies**  01 = Dwelling/house or brick/concrete block structure on a separate stand or yard or on farm  02 = Traditional dwelling/hut/structure made of  traditional materials  03 = Dwelling/house/flat/room in backyard  04 = Informal dwelling/shack in backyard  05 = Informal dwelling/shack not in backyard, e.g. in  an informal/squatter settlement or on farm  06 = Room/flatlet on a property or a larger dwelling/  servants’ quarters/granny flat  07 = Caravan/tent  08 = Other (specify) | **Main Dwelling** | **Other Dwelling** |
| --- | --- | --- |

| **6.2 What is the main material used for the walls and the roof of the main dwelling?**  01 = Bricks  02 = Cement block/concrete  03 = Corrugated iron/zinc  04 = Wood  05 = Plastic  06 = Cardboard  07 = Mud and cement mix  08 = Wattle and daub  09 = Tile  10 = Mud  11 = Thatching/grass  12 = Asbestos  13 = other (specify) | **Walls** | **Roof** |
| --- | --- | --- |

| **6.4** | **What is the household’s main source of drinking**  **water?**  01 = Piped (tap) water in dwelling/house  02 = Piped (tap) water in yard  03 = Borehole in yard  04 = Rain-water tank in yard  05 = Neighbour’s tap  06 = Public/communal tap  07 = Water-carrier/tanker  08 = Borehole outside yard  09 = Flowing water/stream/river  10 = Stagnant water/dam/pool  11 = Well  12 = Spring  13 = Other (specify) |  |
| --- | --- | --- |
|  |  |  |
|  |  |  |

| **WATE** | **R - *Ask all households*** |  |
| --- | --- | --- |
| **6.3** | **What is the household’s main source of water for washing and bathing**  01 = Piped (tap) water in dwelling/house  02 = Piped (tap) water in yard  03 = Borehole in yard  04 = Rain-water tank in yard  05 = Neighbour’s tap  06 = Public/communal tap  07 = Water-carrier/tanker  08 = Borehole outside yard  09 = Flowing water/stream/river  10 = Stagnant water/dam/pool  11 = Well  12 = Spring  13 = Other (specify) |  |
|  |  |  |
|  |  |  |
| ***Ask if w*** | ***Water is not in dwelling, or in yard.*** |  |
| **6.5** | **How far is the water source from the dwelling or yard (200m is equal to the length of two football/soccer fields)?**  1 = Less than 200 metres  2 = 201 - 500 metres  3 = 501 metres - 1 kilometre  4 = More than 1 kilometre  5 = Do not know | 1  2  3  4  5 |
| ***Ask if w*** | ***water is not from a pipe or a tap.*** |  |
| **6.6** | **Did you use piped or tap water at any time in the past while living in this community, but have stopped as a result of the system breaking down?**  1 = Yes  2 = No | 1  2 |
|  | |  |

| **6.7** | **Is the water from the main source of drinking water before any**  **treatment …..**  *Read all the options*  1 = Safe to drink?  2 = Clear (has no colour / free of mud)?  3 = Good in taste?  4 = Free from bad smells? | Yes No  1 2  1 2  1 2  1 2 |
| --- | --- | --- |
| **6.8** | **Do household members treat the water used for drinking?** *This may include boiling, adding chlorine or other chemicals, filtering.*  1 = Yes, always  2 = Yes, sometimes  3 = No, never | 1  2  3 |
| **6.9** | **Does the household pay for water?**  1 = Yes  2 = No | 1  2 |
|  |  |  |

| **6.11** | **Is the toilet facility in the dwelling, in the yard or outside the yard?**  1 = In dwelling  2 = In yard  3 = Outside yard | 1  2  3 |
| --- | --- | --- |
|  |  |  |
|  | |  |

| **SANITATION - *Ask all households*** | | |  |
| --- | --- | --- | --- |
| **6.10** | **What type of toilet facility does this household use?**  1 = Flush toilet connected to a public sewerage system  2 = Flush toilet connected to a septic tank  3 = Chemical toilet  4 = Pit latrine/toilet with ventilation pipe  5 = Pit latrine/toilet without ventilation pipe  6 = Bucket toilet  7 = None  8 = Other (specify) |  |  |
|  |  |  |  |
|  |  |  |  |
|  |  |  | |

| **6.12** | **Does this household have access to/use electricity?**  1 = Yes  2 = No  3 = Do not know | 1  2  3 |
| --- | --- | --- |
|  |  |  |

| **6.13** | **What is the main source of energy/fuel for cooking in this household?**  **01 = Electricity from mains**  **02 = Electricity from generator**  **03 = Gas**  **04 = Paraffin**  **05 = Wood**  **06 = Coal**  **07 = Candles**  **08 = Animal dung**  **09 = Solar energy**  **10 = Other, (specify)**  **11 = None** |  |
| --- | --- | --- |
|  |  |  |

| **7.5** | **In the past 12 months, did any adult (18 years and over) in this**  **household ever go to bed at night without food because of a lack of resources to get food.**  1 = Never  2 = Rarely (1 – 2 times a month)  3 = Sometimes (3 – 10 times a month)  4 = Often (more than 10 times a month)  5 = Always  6 = Not applicable (No adults in household) | No: ___ |
| --- | --- | --- |
| **7.6** | **In the past 12 months, did any child (17 years or younger) in this**  **household ever go to bed at night without food because of a lack of resources to get food.**  1 = Never  2 = Rarely (1 – 2 times a month)  3 = Sometimes (3 – 10 times a month)  4 = Often (more than 10 times a month)  5 = Always  6 = Not applicable (No adults in household) | No: ___ |
| **7.7** | **In the past 12 months, did any child (17 years or younger) in this**  **household ever go a whole day and night without food because of a lack of resources to get food?**  1 = Never  2 = Rarely (1 – 2 times a month)  3 = Sometimes (3 – 10 times a month)  4 = Often (more than 10 times a month)  5 = Always  6 = Not applicable (No adults in household) | No: ___ |
| **7.8** | **In the past 12 months, did any child (17 years or younger) in this**  **household ever go a whole day and night without food because of a lack of resources to get food?**  1 = Never  2 = Rarely (1 – 2 times a month)  3 = Sometimes (3 – 10 times a month)  4 = Often (more than 10 times a month)  5 = Always  6 = Not applicable (No adults in household) | No: ___ |
|  |  |  |

**SECTION 7: HOUSEHOLD** **FOOD SECURITY (caregiver to answer)**

| **7.1** | **In the past 12 months, did any adult (18 years and above) in this**  **household go hungry because of a lack of resources to get food?**  1 = Never  2 = Rarely (1 – 2 times a month)  3 = Sometimes (3 – 10 times a month)  4 = Often (more than 10 times a month)  5 = Always  6 = Not applicable (No adults in household) | No:___ |
| --- | --- | --- |
| **7.2** | **In the past 12 months, did any child (17 years or younger) in this**  **household go hungry because of a lack of resources to get food?**  1 = Never  2 = Rarely (1 – 2 times a month)  3 = Sometimes (3 – 10 times a month)  4 = Often (more than 10 times a month)  5 = Always  6 = Not applicable (No adults in household) |  |
| **7.3** | **In the past 12 months, did any child (17 years or younger) in this**  **household eat less often than you feel they should because of a lack of resources to get food.**  1 = Never  2 = Rarely (1 – 2 times a month)  3 = Sometimes (3 – 10 times a month)  4 = Often (more than 10 times a month)  5 = Always  6 = Not applicable (No adults in household) |  |
| **7.4** | **In the past 12 months, did any child (17 years or younger) in this**  **household eat smaller meals than you feel they should because of a lack of resources to get food.**  1 = Never  2 = Rarely (1 – 2 times a month)  3 = Sometimes (3 – 10 times a month)  4 = Often (more than 10 times a month)  5 = Always  6 = Not applicable (No adults in household) |  |
|  |  |  |
| **7.8** | **In the past 12 months, was there any young person, aged 5 - 17 years, who has left this household, and you do not know his/her whereabouts or to live on the streets?**  1 = Yes  2 = No  3 = Do not know  4 = Not applicable (No children in household) | 1  2  3  4 |
| **7.9** | **Did your household run out of money to buy food during the**  **past 12 months? *If “No” Go to Q7.10***  Has it happened 5 or more days in the past 30 days? | Yes No  1 2  1 2 |
| **7.10** | **Did you cut the size of meals during the past 12 months because**  **there was not enough food in the house?**  ***If “No” Go to Q7.11***  Has it happened 5 or more days in the past 30 days? | Yes No  1 2  1 2 |
|  | |  |

| **7.9** | **Did you skip any meals during the past 12 months because there**  **was not enough food in the house?**  Has it happened 5 or more days in the past 30 days? | Yes No  1 2  1 2 |
| --- | --- | --- |
| **7.10** | **Did you eat a smaller variety of foods during the past 12 months than you would have liked to, because there was not enough food in the house?**  Has it happened 5 or more days in the past 30 days? | Yes No  1 2  1 2 |
| **7.11**  **7.12** | How many times in a typical day do adults eat?  How many times in a typical day do children under 5 years eat? | No____  No____ |
|  |  |  |

| Please describe the foods (meals and snacks) that you ate or drank yesterday during the day and night, whether at home or outside the home. Start with the first food or drink of the morning. *Include foods eaten by any member of the household, and exclude foods that were purchased and eaten outside the home.* | | | | | | |
| --- | --- | --- | --- | --- | --- | --- |
|  | Breakfast  (Morning) | Snack  (In-between) | Lunch  (Midday) | Snack  (In-between) | Dinner  (Evening) | Snack  (Night) |
| **7.13** |  |  |  |  |  |  |

| **8.1** | ***Ask about the food consumption of household members*** | **Did household members eat this food yesterday?** | How often is this food group usually eaten in the household? Think of the past year, and then fill in the number of times: either per week OR per month OR per year. | | | Where was the food obtained from (source)? | | |
| --- | --- | --- | --- | --- | --- | --- | --- | --- |
|  |  |  |  |  |  | Produced (e.g. from own garden) | In kind/donations/  event  Gift/food bank/school feeding | Bought (name store) |
|  |  | Yes - tick | Per week | Per month | Per year |  |  |  |
|  | 01 = Cereals: maize, rice, wheat, sorghum, millet, and any other foods made from cereals such as porridge, bread and noodles |  |  |  |  |  |  |  |
|  | 02 = White roots and tubers - Potatoes, white sweet potato and cassava |  |  |  |  |  |  |  |
|  | 03 = Orange-flesh vegetables: Pumpkin, carrot, butternut or sweet potato |  |  |  |  |  |  |  |
|  | 04 –Dark green leafy vegetables, including wild/indigenous vegetables |  |  |  |  |  |  |  |
|  | 05 – Other vegetables (tomato, onion, green beans, gem squash, eggplant, including wild/indigenous vegetables |  |  |  |  |  |  |  |
|  | 06 - Orange-coloured fruit (e.g. ripe mango, apricot, spanspek, papaya, dried peach and 100% fruit juice made from these)? |  |  |  |  |  |  |  |
|  | 07 - Other fruit (e.g. oranges, banana, apple, pear etc.), including wild/indigenous vegetables? |  |  |  |  |  |  |  |
|  | 08 - Organ meat (liver, kidney, heart or other organ meats or blood-based foods) |  |  |  |  |  |  |  |
|  | 09 - Meat (e.g. beef, goat, sheep, poultry, pork, fish, insects |  |  |  |  |  |  |  |
|  | 10 - Eggs from any animal |  |  |  |  |  |  |  |
|  | **11 -**  Fish and seafood (fresh, tinned or dried and shellfish) |  |  |  |  |  |  |  |
|  | **12 -** Dried beans, peas, lentils, nuts, seeds or foods made from these (e.g. peanut butter)? |  |  |  |  |  |  |  |
|  | **13 -** Milk and milk products (e.g. yoghurt, maas cheese) |  |  |  |  |  |  |  |
|  | **14 -**  Oils and fats(e.g. sunflower, rama, lard, butter added to food or used for cooking |  |  |  |  |  |  |  |
|  | **15 -**  Sweets (e.g. sugar, honey, sweetened juices or fizzy drinks, sugary foods such as chocolate, cookies, cakes) |  |  |  |  |  |  |  |
|  | **16 -**  Spices (e.g. pepper and salt), condiments (e.g. tomato sauce), coffee, tea, alcoholic beverages |  |  |  |  |  |  |  |

| **8.2 Ask to see the food in stock in the household (including on-farm stores such as granaries). Record this below.** | | |
| --- | --- | --- |
| Food item | Unit quantity (size of the package when full) | Quantity in stock |
|  |  |  |
|  |  |  |
|  |  |  |
|  |  |  |
|  |  |  |
|  |  |  |
|  |  |  |
|  |  |  |
|  |  |  |
|  |  |  |
|  |  |  |
|  |  |  |
|  |  |  |
|  |  |  |

**Section 9: Months of Adequate Household Food Provisioning (MAHFP) for Measurement of Household Food Access**

| **9.0** | QUESTIONS AND FILTERS CODING |  |  |
| --- | --- | --- | --- |
| **9.1** | Now I would like to ask you about your household’s food supply during different months of the year. When responding to these questions, please think back over the last 12 months.  **PLACE A *ONE* IN THE BOX IF THE RESPONDENT**  **ANSWERS YES** TO THE FOLLOWING QUESTION.  PLACE A *ZERO* IN THE BOX IF THE RESPONSE IS NO.  In the past 12 months, were there months in which you did not have enough food to meet your family’s needs? [1=Yes, 0=No] |  | IF NO,  END  HERE |
| **9.2** | DO NOT READ THE LIST OF MONTHS.  WORKING BACKWARD FROM THE CURRENT MONTH, **PLACE A ONE IN THE BOX IF THE**  **RESPONDENT IDENTIFIES THAT MONTH AS ONE IN WHICH THE HOUSEHOLD DID NOT HAVE ENOUGH FOOD** TO MEET THEIR NEEDS.  If yes, which were the months (in the past 12 months) in which you did not have enough food to meet your family’s needs? |  |  |
| **A**  **B**  **C**  **D**  **E**  **F**  **G**  **H**  **I**  **J**  **K**  **L** | January  February  March  April  May  June  July  August  September  October  November  December | A……………..  B……………..  C……………..  D……………..  E……………..  F……………..  G……………..  H……………..  I……………..  J……………..  K……………..  L…………….. |  |

| **9.3** How does the household cope with major income shocks (e.g. drought, death of a breadwinner, job loss, etc.) (Please tick where appropriate) | |
| --- | --- |
| Sell livestock | **Y  N** |
| Sell other assets | **Y  N** |
| Use own cash savings | **Y  N** |
| Borrow money from relatives | **Y  N** |
| Borrow money from stokvel | **Y  N** |
| Receive help from friends or relatives | **Y  N** |
| Take on additional work | **Y  N** |
| Reduce spending | **Y  N** |
| Reduce food consumption | **Y  N** |
| Reduce or stop debt repayments | **Y  N** |
| Other: Please specify | **Y  N** |

| **9.4** How much does the household spend on food: | |
| --- | --- |
| Per week? |  |
| Per month? |  |

|  | **Section 10: HOUSEHOLD LIVELIHOODS AGRICULTURAL ACTIVITIES *Ask all households*** |  |
| --- | --- | --- |
| **10.1** | **Has the household been involved in the production of any kind of food or agricultural products during the past twelve months? (e.g. livestock, crops, poultry, food gardening, forestry, fish, etc.)**  1 = Yes  2 = No | 1  2 |
| **10.2** | **What kind of food production/agricultural activities is the**  **household involved in?**  *Read all the options*  01 = Livestock production (cattle, goats, sheep, pigs, etc.)  02 = Poultry production (chickens, ducks, geese, guinea fowl, etc.)  03 = Grains and food crops (maize, wheat, beans, sorghum, millet,  Groundnuts etc.)  04 = Industrial crops (e.g. tea, coffee, cotton, sugar, tobacco)  05 = Fruit and vegetable production  06 = Fodder, grazing/pasture or grass for animals  07 = Fish farming/aquaculture  08 = Forestry  09 = Game farming  10 = Other | Yes No  1 2  1 2  1 2  1 2  1 2  1 2  1 2  1 2  1 2  1 2 |
| **10.3** | **Why do you grow farm produce or keep livestock for the household in the past year?**  1 = As a main source of food for the household  2 = As the main source of income/earning a living  3 = As an extra source of income  4 = As an extra source of food for the household  5 = As a leisure activity or hobby e.g. gardening | 1  2  3  4  5 |
| **10.4** | **Did your household sell any of its produce or livestock in the last year?**  1 = Yes  2 = No | 1  2 |
|  |  |  |

| **10.5** | Do you store any foods for later use? | If yes, what foods? |
| --- | --- | --- |
| **10.6** | Do you process any foods? | If yes, what foods? |

| **10.11** | **How many of the following does the**  **household own?** *Please mark the most appropriate category with an x. Answer if option 1 in Q8.3 = 1.*     1. = Cattle for food or investment 2. = Donkeys and mules   2 = Sheep  3 = Goats  4 = Pigs  5 = Poultry  6 = Other | 0 1-10 11-100 100+ |
| --- | --- | --- |
|  |  |  |

|  |  |  |
| --- | --- | --- |
| **10.7** | **If produce is sold, to whom does your household sell most of its produce?**  *Read all the options*  1 = Local buyers from this district  2 = Buyers from neighboring cities and towns  3 = Formal markets in South Africa  4 = Export agencies in international buyers.  5 = Other | 1  2  3  4  5 |
| **10.8** | **Has your household received any of the following kinds of agricultural related assistance from the government during the past 12 months?**  *Read all the options*  1 = Training  2 = Advice from government extension officers  3 = Grants (money that does not have to be paid back)  4 = Loans (money that has to be paid back)  5 = Inputs (seed, fertilizer, etc.) as part of a loan  6 = Inputs (seed, fertilizer, etc.) for free  7 = Dipping and vaccination services for livestock from State  veterinarian or other Department  8 = Other (specify)  **Go to Q8.6b if households answered yes to any of the**  **categories above, else go to Q8.7** | Yes No  1 2  1 2  1 2  1 2  1 2  1 2  1 2  1 2 |
|  |  |  |
|  |  |  |
| **10.9** | **Did your household find this agriculture-related assistance:**  1 = Very useful  2 = Somewhat useful  3 = Not useful | 1  2  3 |
| 1**0.10** | **Did your household receive agriculture-related assistance from**  **any other entity than government?**  1 = Yes  2 = No | 1  2 |
|  |  |  |

***Continue if the household planted grains/vegetables/fruits/trees (forestry)/pastures/***

***industrial crops. Otherwise, go to Q8.9a***

| **10.12** | **Where does the household practice its crop planting activities?**  *Read all the options*  1 = Farm land (communal or private)  2 = Backyard garden (can include, vegetables, fruits, grains )  3 = School garden (can include, vegetables, fruits, grains)  4 = Communal garden (more than one household involved, can  include vegetables, fruits, grains )  5 = On verges of roads and unused public/municipal land  6 = Other | Yes No  1 2  1 2  1 2  1 2  1 2  1 2 |
| --- | --- | --- |
| **10.13** | **Approximately how big is the land that the household uses for**  **production? Estimate total area if more than one piece.**  1 = Less than 500m2 (approximately one soccer field)  2 = 500m2 to 9 999m2 (between one soccer field and one hectare)  3 = 1 but less than 2 hectares  4 = 2 but less than 5 hectares  5 = 5 but less than 10 hectares  6 = 10 but less than 20 hectares  7 = 20 or more hectares  8 = Do not know |  |
|  | | |

| 1**0.14** | **On what basis does this household have access to the land used for crop production? If more than one kind of tenure system applies for different pieces of land, give an answer for the**  **biggest piece.**  1 = Owns the land  2 = Rents the land  3 = Sharecropping  4 = Tribal authority  5 = State land  6 = Other (specify)  7 = Do not know |  |
| --- | --- | --- |
|  |  |  |
|  |  |  |
|  |  |  |

| 1**0.15** | **If the household receives an income from remittances, please specify approximately how much they receive per month?** *If no income received from remittances write 0.* |  | | | |
| --- | --- | --- | --- | --- | --- |
| **10.16** | **If the household receives an income from pensions (do not include income from old age grants), please specify approximately how much they receive per month?** *If no income received from pensions, write 0.* |  | | | |
|  |  |  |  |  |  |
|  |  |  |  |  |  |
|  |  |  | | | |
| **10.17** | **What was the total household expenditure in the last month?** *Include money spent on food, clothing, transport, rent and rates, alcohol and tobacco, school fees, entertainment and any other expenses.* |  | | | |
|  |  |  | | | |
|  |  |  | | | |

| 1**0.18** | **How many of the following does the household own?**  01 = Bed with mattress  02 = Sofa Set  03 = Table (dining/desk)  04 = Pay TV (M-Net / DSTV / Top TV) Subscription  05 = Radio-working condition  06 = Mobile Phone  07 = Tape or CD/DVD  08 = Television  09 = Motor vehicle  10 = Refrigerator  11 = Washing machine  12 = Electric Stove / Gas Stove | Number  01 =  02 =  03 =  04 =  05 =  06 =  07 =  08 =  09 =  10 =  01 =  11 =  12= |
| --- | --- | --- |
|  |  | |

**SECTION 11: CROPS PRODUCED in the 2012/13 season (X month 2012 to this month 2013)**

| **11.1** | Crops grown | Month 1 | Month 2 | Month 3 | Month 4 | Month 5 | Month 6 | Month 7 | Month 8 | Month 9 | Month 10 | Month 11 | Month 12 |
| --- | --- | --- | --- | --- | --- | --- | --- | --- | --- | --- | --- | --- | --- |
|  |  |  |  |  |  |  |  |  |  |  |  |  |  |
|  |  |  |  |  |  |  |  |  |  |  |  |  |  |
|  |  |  |  |  |  |  |  |  |  |  |  |  |  |
|  |  |  |  |  |  |  |  |  |  |  |  |  |  |

| Crops planted and harvested | Total area planted | Total harvest | Price per unit | Did the household consume this crop? | Was any sold? Where? | Income | Was this processed in any way? | How much was stored for home consumption? | Was the crop irrigated? | If irrigated, what kind of irrigation?   - buckets form a river, - treadle pump, - flood irrigation - irrigation scheme, - municipal water | If irrigated, what is the water source? | Did you use any inputs?  Improved seeds?  Fertilizer?  Manure? | Is mechanized farming used?  Tractor?  Harvester? |
| --- | --- | --- | --- | --- | --- | --- | --- | --- | --- | --- | --- | --- | --- |
|  |  |  |  |  |  |  |  |  |  |  |  |  |  |
|  |  |  |  |  |  |  |  |  |  |  |  |  |  |
|  |  |  |  |  |  |  |  |  |  |  |  |  |  |
|  |  |  |  |  |  |  |  |  |  |  |  |  |  |

| **11.2** |  |  |  |
| --- | --- | --- | --- |
|  | Do you apply crop rotation? Y/N | **1=Yes 2=No** | **Why?** |
|  | Is there un-used land? Why? | **1=Yes 2=No** | **Why?** |
|  | Is there follow land? Why? | **1=Yes 2=No** | **Why?** |
|  | Do you have access to water? | **1=Yes 2=No** | **Why?** |
|  | Do you use it for irrigation? | **1=Yes 2=No** | **Why?** |
|  | Do you have enough water? | **1=Yes 2=No** | **Why?** |
|  | What are you not growing and would like to? |  |  |
|  | Have you had poor success with a crop? Why? | **1=Yes 2=No** | **Why?** |

**Thank the respondent!**

**INTERVIEW END TIME: -------------------------**
